# Supplementary figures and images for: Somatic Mutation of the Non-Muscle-Invasive Bladder Cancer Associated with Early Recurrence
Source: Diagnostics (Basel). 2023 Oct 13;13(20):3201. doi: 10.3390/diagnostics13203201 (PMC10606398; doi:10.3390/diagnostics13203201)

group relapse primary

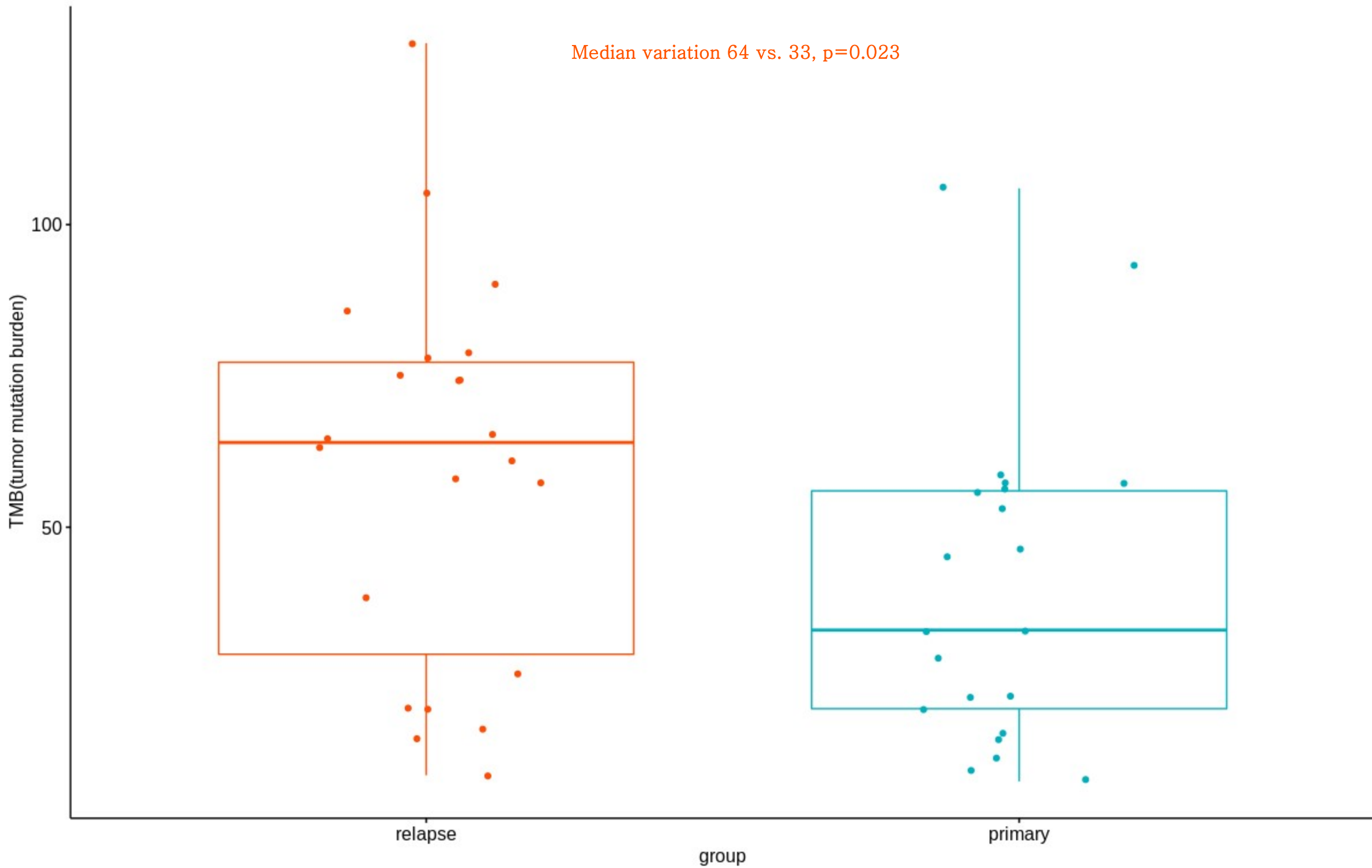

Supplement: Supplementary file 1 [file diagnostics-13-03201-s001.zip › diagnostics-2587484-supplementary.pdf]
